# Exploration of the Role of Cilostazol in Brugada Syndrome: Mechanisms, Therapeutic Potential, and Implications in the Prevention of Ventricular Arrhythmias

**DOI:** 10.31083/RCM43173

**Published:** 2026-01-16

**Authors:** Mohammad Iqbal, Iwan Cahyo Santosa Putra, Giky Karwiky, Chaerul Achmad

**Affiliations:** ^1^Department of Cardiology and Vascular Medicine, Faculty of Medicine, Padjadjaran University, 40161 Bandung, Indonesia

**Keywords:** Brugada syndrome, ventricular arrhythmia, sudden cardiac death, pharmacology, cilostazol

## Abstract

Despite the relatively low incidence of Brugada syndrome (BrS) globally, the risk of sudden cardiac death remains alarmingly high, reaching rates of up to 28%. According to current clinical guidelines, implantable cardioverter defibrillators (ICDs) are recommended for high-risk patients. Meanwhile, pharmacological interventions must be used as a backup owing to the limited access to ICDs by eligible patients. Cilostazol, an adenosine uptake inhibitor and phosphodiesterase III inhibitor, has been suggested to reduce the risk of ventricular arrhythmias in BrS patients by stabilizing the action potential dome and lowering the epicardial-to-endocardial repolarization gradient, consequently decreasing the probability of phase II re-entry. However, the effectiveness of cilostazol in this situation has been questioned due to the existence of contradictory results from different case reports. Thus, this literature review aims to synthesize current evidence regarding the potential of cilostazol to lower the risk of ventricular arrhythmias in patients with BrS.

## 1. Introduction

Brugada syndrome (BrS) is an autosomal dominant genetic condition characterized by coved ST-segment elevation and T-wave inversion in the right precordial leads, increasing the risk of sudden cardiac death (SCD) from ventricular tachyarrhythmia [1]. A meta-analysis conducted by Vutthikraivit *et al*. [2] indicated a worldwide prevalence of BrS at 0.5 per 1000 individuals (95% CI: 0.3–0.7), with the greatest prevalence recorded in Southeast Asia (3.7 per 1000, 95% CI: 0.7–6.7) and the lowest in North Africa (0 per 1000). Despite its low incidence, BrS constitutes up to 28% of sudden cardiac death cases [3]. Numerous factors of sudden cardiac death in Brugada syndrome patients have been found and verified using meta-analyses. These encompass spontaneous coved-type ST-segment elevation in the right precordial leads [type I Brugada electrogram (ECG) pattern], a history of syncope, affirmative electrophysiological studies, and distinct electrocardiographic parameters (e.g., first-degree atrioventricular block, fragmented QRS, wide QRS complex, S wave in lead I, aVR sign, early repolarization in the inferolateral region, atrial fibrillation, Tpeak-Tend dispersion, Tpeak-Tend interval, and the (Tpeak-Tend)/QTc ratio) [4, 5, 6, 7, 8, 9, 10].

The American Heart Association (AHA) and the European Society of Cardiology (ESC) have recently recommended that high-risk BrS patients have implanted cardioverter-defibrillators (ICDs); however, access to ICDs is still limited, particularly in the least developed countries [11, 12]. Only 12% of 3240 ICD-eligible patients in Asia who participated in the survey had received an ICD. Indonesia used ICDs at the lowest rate of 1.5%, while Japan used them at the highest rate of 52.5% [13]. In these settings, pharmaceutical treatment emerges as a vital option. At present, Quinidine is the most well researched antiarrhythmic agent for BrS and has class IIa recommendations for pharmacological intervention [12]. Nonetheless, the accessibility of these medications remains constrained. In contrast, cilostazol, a phosphodiesterase III and adenosine uptake inhibitor, is an accessible medication that has been suggested as a possible option for mitigating arrhythmogenesis in BrS patients [14]. Despite its promise, research on cilostazol has shown contradictory findings about its efficacy in reducing ventricular arrhythmias in BrS patients [15, 16, 17, 18]. Moreover, existing clinical recommendations do not yet recognize cilostazol as a recommended therapeutic choice for BrS [11, 12]. This literature review seeks to synthesize the existing information about the pleiotropic effects of cilostazol and its mechanisms of action in mitigating the risk of sudden cardiac death in individuals with BrS.

## 2. Pharmacokinetics of Cilostazol

Cilostazol is a potent and targeted phosphodiesterase (PDE) 3A inhibitor. When taken orally, it is easily absorbed, and when taken with a high-fat meal, absorption is greatly enhanced. The drug has uneven absorption and is primarily broken down by Cytochrome P450 3A4 (CYP3A4) and Cytochrome P450 3A5 (CYP3A5) in the liver, with Cytochrome P450 2C19 (CYP2C19) playing a minor role. Active metabolites 3,4-dehydrocilostazol (OPC-13015) and 4^′^-trans-hydroxy-cilostazol (OPC-13213) are produced by this metabolic process [19]. With extended treatment, the active metabolites of cilostazol nearly double their baseline levels, reaching steady-state concentrations after a few days. Its half-life is approximately 10 to 13 hours. Cilostazol usually has a therapeutic plasma concentration of 3 ± 5 µM. The kidneys remove most metabolites, accounting for roughly 75% of total drug clearance [20].

Mallikaarjun *et al*. [21] did an experimental study to see how cilostazol affected people with very bad kidney problems (creatinine clearance 0.3 ± 1.6 L/h (5 ± 25 mL/min)). On days 1 and 8, participants took 50 mg of cilostazol every day. On days 2 through 7, they took it every other day. The results showed that the Cmax and AUC0-12h values were 29% and 39% lower, respectively, than those of healthy people. However, these differences were not statistically significant (*p* = non-significant). The drug concentrations in different groups with different renal functions were not very different. This means that cilostazol and its metabolites work the same way no matter how well your kidneys work [21].

Bramer and Forbes [22] did a randomized controlled study to look at how a single 100 mg dose of cilostazol affected the pharmacokinetics in 12 patients with liver problems (10 with mild problems and 2 with severe problems) and 12 healthy controls. The study found that there was not much of a difference in protein binding between healthy people and people with liver problems (95.2% vs. 94.6%). Compared to controls, patients with hepatic dysfunction had much lower medication clearance and total urine metabolite excretion. The pharmacokinetics of cilostazol and its metabolites in people with mild to moderate liver damage were similar to those in healthy people, which means that this group does not need to change their doses. However, doctors should be careful when giving cilostazol to people with moderate to severe liver damage [22].

## 3. Pharmacodynamics of Cilostazol

**Fig. 1.**
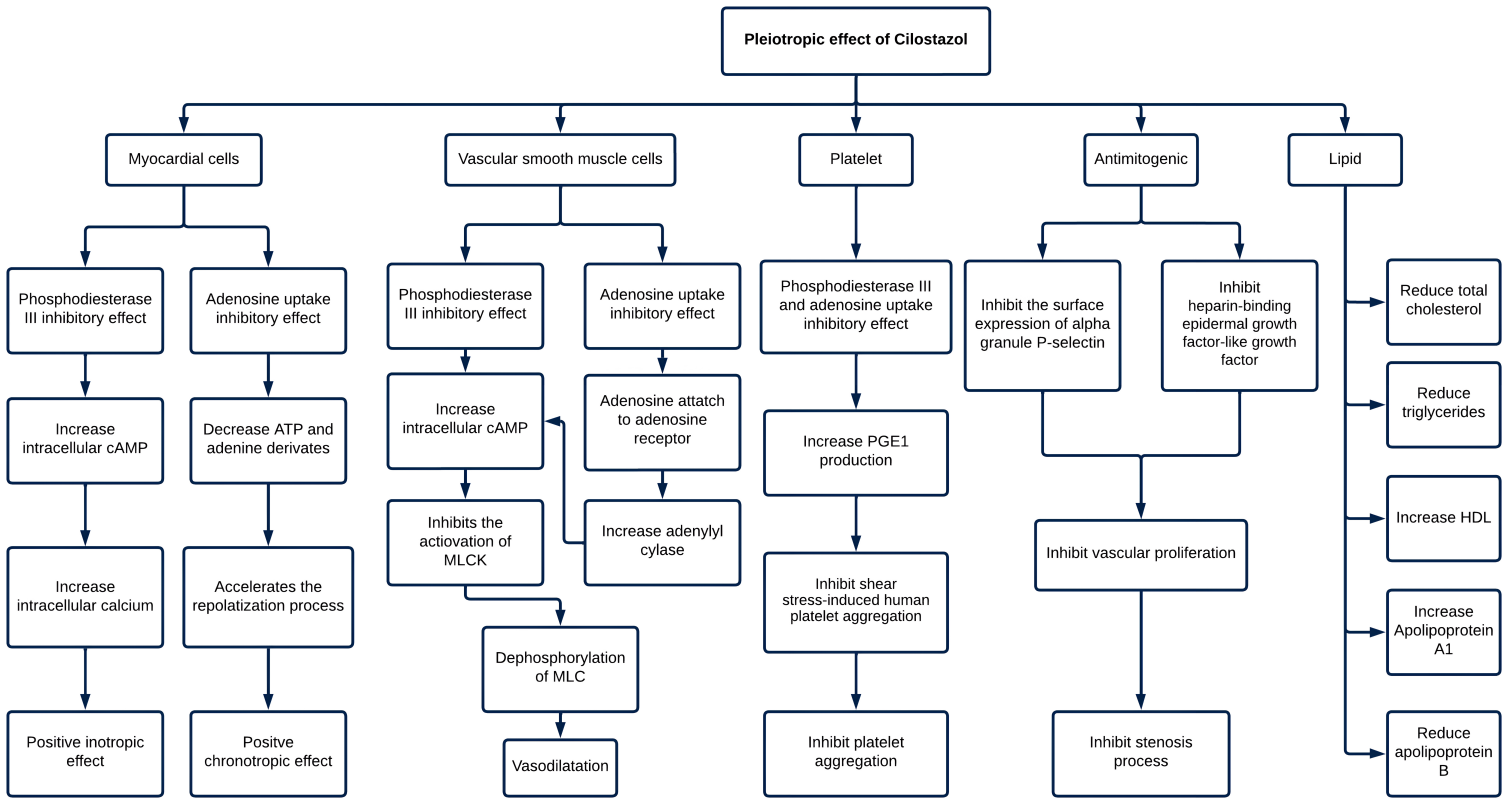
**The pleiotropic effect of cilostazol**. cAMP, cyclic adenosine monophosphate; ATP, adenosine triphosphate; MLCK, myosin light chain kinase; MLC, myosin light chains; PGE1, prostaglandin E1; HDL, high-density lipoprotein.

Cilostazol is a potent and selective inhibitor of phosphodiesterase III, which suppresses the degradation of cyclic adenosine monophosphate (cAMP). This inhibition leads to increased cAMP levels in platelets, cardiomyocytes, and vascular smooth muscle cells, resulting in platelet aggregation inhibition, enhanced inotropic and chronotropic effects, and vasodilation, respectively [21]. Additionally, cilostazol exhibits pleiotropic effects, including lipid-modulating and antimitogenic properties [20]. Reported side effects of cilostazol include headache, diarrhea, palpitations, and edema. However, while there is a theoretical risk of bleeding associated with cilostazol, studies have demonstrated no significant difference in bleeding risk between the cilostazol group and the control group [23]. The detailed mechanisms of cilostazol’s actions are outlined in Fig. 1.

## 4. Effect of Cilostazol on Cardiomyocytes

By stopping phosphodiesterase III, cilostazol raises cAMP levels in cardiomyocytes. More cAMP acts as a second messenger, causing protein kinase A (PK-A) to add phosphate groups to the sarcoplasmic reticulum and L-type calcium channels. This causes calcium to leave the sarcoplasmic reticulum and enter the cell, which raises the amount of calcium inside the cell and has a positive inotropic effect [20]. Cone *et al*. [24] found that cilostazol stops adenosine from being taken up by cardiomyocytes, smooth muscle cells in the coronary arteries, and endothelial cells. The median effective dose was 10 µM. This lowers the levels of intracellular adenosine triphosphate and adenine derivatives, which has a positive effect on the heart’s rhythm [24].

## 5. Effect of Cilostazol on Vascular Smooth Muscle Cells 

Phosphodiesterase III inhibition and adenosine reuptake inhibition are the two ways that ciclostazol produces its vasodilatory effects. Inhibition of phosphodiesterase III raises cAMP levels in vascular smooth muscle cells, which in turn reduces the activity of myosin light chain kinase (MLCK). Myosin light chains (MLC) are dephosphorylated as a result of this inhibition, which lessens vascular smooth muscle contraction and increases vasodilation. Further adenosine binding to its receptors on vascular smooth muscle cells is facilitated by the adenosine reuptake inhibitory effect, which also increases cAMP production and adenylyl cyclase activity, leading to further vasodilation [20].

## 6. Other Pleiotropic Effects of Cilostazol 

Cilostazol elevates cAMP levels in platelets by inhibiting phosphodiesterase III and adenosine absorption, hence augmenting prostaglandin E1 (PGE1) synthesis and mitigating shear stress-induced platelet aggregation (SIPA) [20, 25, 26].

Cilostazol also favourably affects plasma lipoproteins by reducing total cholesterol, triglycerides, lipoprotein(a), and apolipoprotein B, while increasing high-density lipoprotein (HDL) and apolipoprotein A1, without affecting low-density lipoprotein levels [27, 28]. Lastly, cilostazol efficiently reduces vascular stenosis by targeting P-selectin and heparin-binding epidermal growth factor-like growth factor (HB-EGF), which are critical mediators of mitogenesis and vascular proliferation [19, 29, 30].

## 7. Drug Interactions of Cilostazol

When used in conjunction with standard antiplatelet medication, cilostazol provides significant benefits without raising the risk of bleeding. According to a number of studies, cilostazol and other antiplatelet medications do not substantially raise the risk of bleeding; instead, they lower the risk of ischemic events in patients with peripheral artery disease, coronary artery disease, and stroke [31, 32, 33, 34, 35, 36]. The combination of cilostazol and a prostacyclin analog synergistically enhances vasodilatory effects and significantly improves the ankle-brachial index in patients with intermittent claudication [37, 38]. Concurrent use of cilostazol with omeprazole and warfarin is considered safe [39, 40]. Erythromycin co-administration may greatly boost cilostazol’s efficacy [41].

## 8. The Pathogenesis of Brugada Syndrome

Right bundle branch block, ST-segment elevation, and an elevated risk of sudden cardiac death are the hallmarks of BrS, which were initially discovered by the Brugada brothers in 1992 [42]. Asymptomatic presentations to syncope or sudden cardiac death are among the clinical manifestations of BrS. Coved ST-segment elevation with T-wave inversion in the right precordial leads is the defining ECG characteristic of BrS [1].

The pathophysiology of BrS has been linked to loss-of-function mutations in the Sodium Voltage-Gated Channel Alpha Subunit 5 (*SCN5A*) gene, which codes for the α-subunit of the NaV1.5 sodium channel. The inward sodium current’s peak is lowered and the upstroke of phase 0 of the cardiac action potential is slowed when the NaV1.5 sodium channel malfunctions, causing delayed activation and premature inactivation [43].

Additional genetic mutations, including those in Sodium Voltage-Gated Channel Alpha Subunit-10 (*SCN10A*) [44, 45], Sodium Voltage-Gated Channel Beta Subunit 2 (*SCN2B*) [46], Potassium Voltage-Gated Channel Subfamily D Member 3 (*KCND3*) [47], and Calcium Voltage-Gated Channel Auxiliary Subunit Alpha2delta 1 (*CACNA2D1*) [48] have also been associated with BrS. These genes encode the α-subunit of the NaV1.8 sodium channel, the β-subunit of the NaV1.5 sodium channel, the transient outward potassium current (Ito), and the late calcium current (ICa), respectively. The net result of these mutations is a reduction in inward sodium and calcium currents, coupled with an increase in transient outward potassium current, which contributes to the electrophysiological abnormalities observed in BrS [1].

The repolarization hypothesis and the depolarization hypothesis are the two primary theories proposed to explain the cellular mechanisms behind BrS [49, 50]. According to the repolarization hypothesis, BrS results from an imbalance between the transient outward potassium current (Ito) and the inward sodium (INa) and calcium (ICa) currents, particularly in the right ventricle’s epicardial layer as opposed to the endocardial layer. The action potential dome is lost in the ventricular epicardium but remains intact in the endocardium due to the observed imbalance, which causes a noticeable notch during phase 1 of the action potential in the epicardium. This phenomenon raises the risk of phase II reentry and ventricular tachyarrhythmia by causing ST-segment elevation and transmural dispersion of repolarization (epicardial-endocardial gradient) [49, 50, 51].

According to the depolarization hypothesis, conduction delays in the right ventricular outflow tract (RVOT) are caused by a decrease in inward sodium current, which leads to BrS. Late potentials found in BrS patients are often associated with conduction delay. It is thought that the conduction heterogeneity seen in the RVOT plays a major role in arrhythmogenesis [49, 50].

The majority of ventricular arrhythmias in BrS patients occur at night, suggesting that nighttime sympathovagal imbalance plays a major role in their occurrence. Increased vagal tone and decreased sympathetic tone are characteristics of the imbalance [52, 53].

This hypothesis is supported by a study by Krittayaphong *et al*. [53], which found that, in comparison to controls, BrS patients had significantly different nighttime average heart rates, average standard deviation of normal RR intervals (ASDNN), and standard deviation of normal RR intervals (SDNN). This indicates that there was a notable sympathovagal imbalance during the night, as evidenced by the decreased heart rate variability seen in BrS patients.

According to an experimental study by Wichter *et al*. [54], 47% of BrS patients had reduced uptake of the norepinephrine analogue [123I]m-iodobenzylguanidine (123I-MIBG), particularly in the inferior and septal regions of the left ventricular wall. This observation suggests decreased sympathetic activity and impaired presynaptic adrenergic function [54]. Krittayaphong *et al*. [53] observed that the low-frequency component of heart rate variability in BrS patients at night was significantly lower than that in asymptomatic BrS patients and controls (2.77 ± 0.4 vs. 3.02 ± 0.3 vs. 3.04 ± 0.3, *p* = 0.024), indicating reduced sympathetic activation in BrS patients. According to the results, a marked reduction in adrenergic activity at night causes a marked increase in sympathovagal imbalance in BrS.

Enhanced vagal tone results in increased acetylcholine synthesis, subsequently activating inhibitory G proteins. This activation inhibits adenylate cyclase activity, leading to a decrease in intracellular cAMP concentrations [55, 56, 57]. Reduced sympathetic activation concurrently inhibits phospholipase C-coupled G proteins, leading to decreased phospholipase C activity and a reduction in the conversion of phosphatidylinositol 4,5-bisphosphate (PIP2) to inositol 1,4,5-trisphosphate (IP3). This restricts calcium release from the sarcoplasmic reticulum. Furthermore, diminished sympathetic stimulation leads to a decrease in stimulatory G protein activity, which further inhibits adenylate cyclase and results in reduced intracellular cAMP levels [55, 56]. Reduced intracellular cAMP levels in myocardial cells decrease calcium influx, leading to a diminished action potential dome in the right ventricular epicardium. This establishes a transmural voltage gradient that enhances ST-segment elevation in BrS patients, consequently heightening their vulnerability to ventricular arrhythmia [58].

## 9. Mechanisms Underlying Cilostazol’s Role in Mitigating Ventricular Arrhythmia Risk in Brugada Syndrome

Cilostazol is essential in mitigating the risk of ventricular arrhythmia in patients with BrS due to its phosphodiesterase III inhibitory properties. Cilostazol increases intracellular cAMP levels, thereby enhancing calcium release from the sarcoplasmic reticulum and facilitating calcium influx into myocardial cells. This process increases intracellular calcium levels, reinstating the action potential dome in right ventricular epicardial cells, which normalizes the ST segment and suppresses ventricular arrhythmias [20, 52, 59]. The experimental study by Szél *et al*. [14] validated this hypothesis, demonstrating that cilostazol restored the action potential dome in the right ventricular epicardium and reduced ST-segment elevation. This effect inhibited phase 2 reentry and successfully prevented ventricular tachycardia/ventricular fibrillation (VT/VF) in all examined right ventricular preparations (6 of 6 preparations, 100%) [14]. Similarly, a case report by Ağaç *et al*. [18] reported that cilostazol administration in a BrS patient led to the spontaneous conversion of a type I Brugada ECG pattern to a type III pattern by reducing ST-segment elevation in the right precordial leads, successfully preventing ventricular arrhythmias during a 10-month follow-up.

Cilostazol also inhibits adenosine uptake, leading to reduced levels of adenosine triphosphate (ATP) and adenine derivatives in myocardial cells. The reduction activates ATP-sensitive potassium channels (IK, ATP), thereby accelerating repolarization. This leads to positive chronotropic effects and an increase in heart rate [20, 52, 59]. Lee *et al*. [60] presented a case report indicating that overdrive pacing at a rate of 90 beats per minute with a dual-chamber implantable cardioverter-defibrillator effectively suppressed ST-segment elevation in a patient with Brugada syndrome, thereby preventing ventricular arrhythmias over an 8-month follow-up period.

**Fig. 2.**
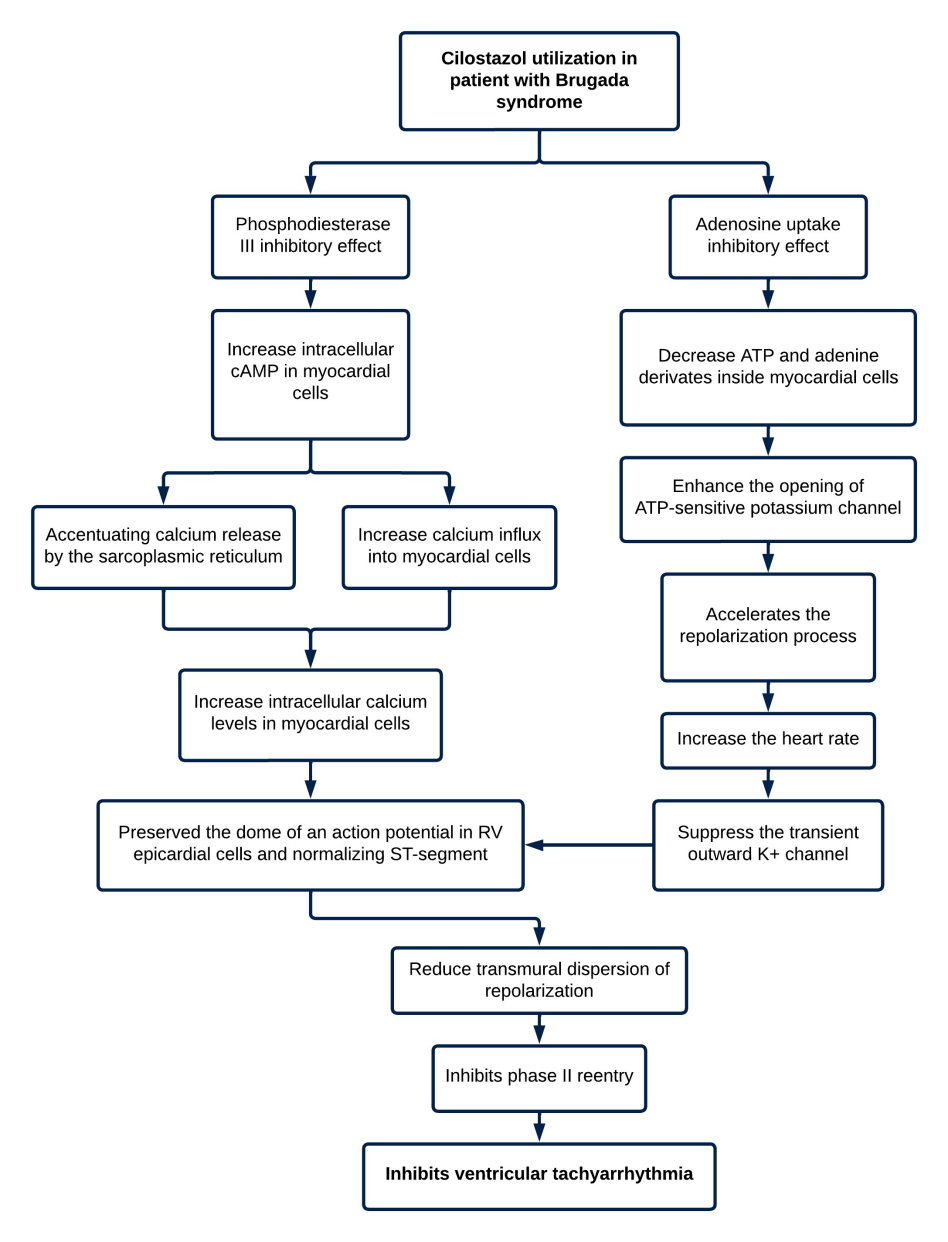
**Mechanism of action of cilostazol in Brugada syndrome**. cAMP, cyclic adenosine monophosphate; ATP, adenosine triphosphate; RV, right ventricle.

Cilostazol’s positive chronotropic effect theoretically inhibits the transient outward potassium current (Ito), thereby reducing ST-segment elevation and the risk of ventricular arrhythmia in BrS patients [20, 52, 59]. Case reports by Tsuchiya *et al*. [17] and Ağaç *et al*. [18] confirmed that cilostazol administration resulted in an increase in heart rate of approximately 10 and 20 beats per minute, respectively. Cilostazol effectively prevented ventricular arrhythmias during follow-up periods of 13 and 10 months, respectively [17, 18]. Fig. 2 illustrates the detailed mechanism of cilostazol’s action in BrS.

## 10. Case Reports on the Use of Cilostazol in Brugada Syndrome Patients

Currently, four case reports have documented the application of cilostazol in patients with BrS, as outlined in Table 1 (Ref. [15, 16, 17, 18]). Two case reports indicated successful termination of ventricular tachyarrhythmia after administering cilostazol at a dosage of 100 mg twice daily [17, 18]. Tsuchiya *et al*. [17] found that episodes of ventricular fibrillation (VF) continued despite administration of a low dose of cilostazol (100 mg/day). Increasing the dose to 200 mg/day led to the cessation of syncope and VF episodes during a 13-month follow-up period [17]. Ağaç *et al*. [18] reported that cilostazol administered at a dosage of 200 mg/day effectively prevented syncope and ventricular fibrillation episodes for a duration of 10 months.

**Table 1.** **Case reports of cilostazol utilization in patients with Brugada syndrome**.

Author (year), country	Patient profile	High risk features of SCD	Management	Outcomes
Tsuchiya *et al*. (2002), Japan [17]	A 67-year-old man with frequent episodes of convulsion during sleep and no structural heart disease was detected	-Syncope	-ICD implantation	-Five episodes of VF within 9 days following ICD implantation prior cilostazol administration
		-Type I Brugada ECG pattern	-Cilostazol 200 mg/day	
		-Positive EPS		-Recurrence of VF episodes after 5 days of administration of low-dose cilostazol (100 mg/day)
				-No syncope and No recurrence VF episodes and shock delivery from the ICD during the follow-up of within 13 months of administration of cilostazol 200 mg/day
Abud *et al*. (2006), Argentina [16]	A 30-year-old man with recurrent episodes of syncope	-Syncope	-ICD implantation	Four episodes of VF after 11 months of administration of cilostazol 200 mg/day
		-Type I Brugada ECG pattern	-Cilostazol 200 mg/day	
		-Atrial fibrillation		
Ağaç *et al*. (2014), Turkey [18]	A 26-year-old man with recurrent episodes of syncope and no structural heart disease was detected	-Syncope	-ICD implantation	-Four episodes of polymorphic VT within one week following ICD implantation
		-Type I Brugada ECG pattern	-Cilostazol 100 mg twice daily	-The ECG was converted from type I to type III Brugada ECG pattern and the disappearance of early repolarization pattern in lateral leads after 2 days of administration of cilostazol 100 mg twice daily
		-Early repolarization pattern in lateral leads		
				-No syncope and no recurrence of VF episodes and ICD shock within 10 months of administration of cilostazol 100 mg twice daily
Shenthar *et al*. (2017), India [15]	A 54-year-old man with recurrent syncope	-Syncope	-ICD implantation	-One episode of VF dan 9 episodes of VT within four years of administration of cilostazol 100 mg twice daily
		-Type I Brugada ECG pattern	-Cilostazol 100 mg twice daily	
		-Atrial fibrillation	-Oral quinine 300 mg thrice daily	-Two episodes of monomorphic VT and 5 episodes of non-sustained polymorphic VT after 3 months of administration of combination of cilostazol 100 mg twice daily and oral quinine 300 mg thrice daily
				-No syncope and no recurrent VT/VF after 4 months of administration of oral quinine 300 mg thrice daily alone

ECG, electrocardiography; ICD, implantable cardioverter defibrillator; EPS, electrophysiology study; VT, ventricular tachycardia; VF, ventricular fibrillation; mg, milligram.

All patients in these reports demonstrated high-risk characteristics for significant arrhythmic events, including syncope and type I Brugada ECG patterns. Two patients reported by Abud *et al*. [16] and Shenthar *et al*. [15] experienced malignant arrhythmias despite receiving cilostazol. Both patients exhibited atrial fibrillation (AF), which serves as an additional risk factor for significant arrhythmic events. Kewcharoen *et al*. [6] conducted a meta-analysis revealing that AF significantly increased the risk of major arrhythmic events in BrS patients, with a pooled odds ratio of 2.37 (95% confidence interval: 1.36–4.13, *p* = 0.002, I^2^ = 40.3%). Additionally, case series conducted by Iqbal *et al*. [61] and Letsas *et al*. [62] demonstrated that BrS patients continued to face an increased risk of sudden cardiac death, even after conversion of AF to sinus rhythm. Multiple hypotheses have been suggested to elucidate the relationship between atrial fibrillation and ventricular arrhythmias in Brugada syndrome. Initially, atrial fibrillation may decrease refractoriness in ventricular muscle, promoting rapid ventricular rates that can lead to ventricular tachyarrhythmia. Secondly, atrial fibrillation’s irregular rhythm may provoke proarrhythmic effects via a short-long-short electrical pattern [63, 64]. Third, particular mutations in the *SCN5A* gene at the atrial level may predispose patients with Brugada syndrome to both ventricular tachycardia and atrial fibrillation, thereby elevating the risk of sudden cardiac death [65].

Cilostazol has been proposed to reveal hidden atrial fibrillation. A randomized controlled trial conducted by Aoki *et al*. [66] indicated that cilostazol serves as a significant and independent predictor of new-onset atrial fibrillation (OR 2.672, 95% CI: 1.205–5.927, *p* = 0.016). Cilostazol elevates intracellular cyclic adenosine monophosphate (cAMP) levels, resulting in increased intracellular calcium concentrations. This mechanism enhances sinus node automaticity and elevates heart rate, which may activate latent atrial fibrillation. AF was observed in patients reported by Abud *et al*. [16] and Shenthar *et al*. [15] after cilostazol administration. This elucidates the increased risk of ventricular arrhythmia and the restricted effectiveness of cilostazol in such instances.

Additional factors may also influence the risk of ventricular arrhythmias in patients with Brugada syndrome. Genotypic variations can lead to unique electrophysiological substrates that exhibit varying sensitivities to antiarrhythmic drugs [67, 68]. Since cilostazol primarily exerts its effects through calcium channels, BrS patients with SCN5A mutations, which predominantly impair sodium current, may not experience the same therapeutic benefit as those with calcium channel mutations, such as those involving the *CACNA2D1* gene. Therefore, genetic testing in BrS patients should be considered in future studies evaluating the efficacy of cilostazol in preventing ventricular arrhythmias in this population. Furthermore, autonomic influences and various biological factors, including body temperature, can induce dynamic alterations in the arrhythmic substrate of BrS patients [69, 70].

## 11. Conclusion

Cilostazol is an accessible medication characterized by its pleiotropic effects and limited drug interactions. In patients with BrS, the dual mechanisms of adenosine uptake inhibition and phosphodiesterase III inhibition are linked to a lower risk of ventricular arrhythmias. Cilostazol has been shown in two case reports to effectively prevent ventricular arrhythmias in Brugada syndrome patients. According to two more case reports, cilostazol does not prevent ventricular arrhythmias because it may reveal latent atrial fibrillation, increasing the risk of arrhythmic events in patients with BrS. When ICD and quinidine are unavailable or contraindicated, 100 mg of cilostazol twice daily may be used as a therapeutic alternative for BrS patients; however, each patient’s risks and benefits must be carefully considered. Further observational studies and randomized controlled trials are required to validate cilostazol’s effectiveness in decreasing the risk of ventricular arrhythmia in BrS patients.

## References

[1] Krahn AD, Behr ER, Hamilton R, Probst V, Laksman Z, Han HC (2022). Brugada Syndrome. *JACC. Clinical Electrophysiology*.

[2] Vutthikraivit W, Rattanawong P, Putthapiban P, Sukhumthammarat W, Vathesatogkit P, Ngarmukos T (2018). Worldwide Prevalence of Brugada Syndrome: A Systematic Review and Meta-Analysis. *Acta Cardiologica Sinica*.

[3] Papadakis M, Papatheodorou E, Mellor G, Raju H, Bastiaenen R, Wijeyeratne Y (2018). The Diagnostic Yield of Brugada Syndrome After Sudden Death With Normal Autopsy. *Journal of the American College of Cardiology*.

[4] Achmad C, Kamarullah W, Putra ICS, Firmansyah DK, Iqbal M, Karwiky G (2023). Investigation of High-Risk Electrocardiographic Markers as Predictors of Major Arrhythmic Events in Brugada Syndrome: A Systematic Review and Meta-analysis. *Current Problems in Cardiology*.

[5] Iqbal M, Putra ICS, Pranata R, Budiarso MN, Pramudyo M, Goenawan H (2022). Electrocardiographic Markers Indicating Right Ventricular Outflow Tract Conduction Delay as a Predictor of Major Arrhythmic Events in Patients With Brugada Syndrome: A Systematic Review and Meta-Analysis. *Frontiers in Cardiovascular Medicine*.

[6] Kewcharoen J, Rattanawong P, Kanitsoraphan C, Mekritthikrai R, Prasitlumkum N, Putthapiban P (2019). Atrial fibrillation and risk of major arrhythmic events in Brugada syndrome: A meta-analysis. *Annals of Noninvasive Electrocardiology: the Official Journal of the International Society for Holter and Noninvasive Electrocardiology, Inc*.

[7] Tse G, Gong M, Li CKH, Leung KSK, Georgopoulos S, Bazoukis G (2018). Tp⁢e⁢a⁢k-Te⁢n⁢d, Tp⁢e⁢a⁢k-Te⁢n⁢d/QT ratio and Tp⁢e⁢a⁢k-Te⁢n⁢d dispersion for risk stratification in Brugada Syndrome: A systematic review and meta-analysis. *Journal of Arrhythmia*.

[8] Chiotis S, Pannone L, Doundoulakis I, Della Rocca DG, Zafeiropoulos S, Sorgente A (2024). Spontaneous type 1 ECG and arrhythmic risk in Brugada syndrome: A meta-analysis of adjusted time-to-event data. *Heart Rhythm O2*.

[9] Rattanawong P, Kewcharoen J, Yinadsawaphan T, Fatunde OA, Kanitsoraphan C, Vutthikraivit W (2023). Type of syncope and outcome in Brugada syndrome: A systematic review and meta-analysis. *Journal of Arrhythmia*.

[10] Bazoukis G, Chung CT, Vassiliou VS, Sfairopoulos D, Lee S, Papadatos SS (2023). The Role of Electrophysiological Study in the Risk Stratification of Brugada Syndrome. *Cardiology in Review*.

[11] Al-Khatib SM, Stevenson WG, Ackerman MJ, Bryant WJ, Callans DJ, Curtis AB (2018). 2017 AHA/ACC/HRS Guideline for Management of Patients With Ventricular Arrhythmias and the Prevention of Sudden Cardiac Death: A Report of the American College of Cardiology/American Heart Association Task Force on Clinical Practice Guidelines and the Heart Rhythm Society. *Circulation*.

[12] Zeppenfeld K, Tfelt-Hansen J, de Riva M, Winkel BG, Behr ER, Blom NA (2022). 2022 ESC Guidelines for the management of patients with ventricular arrhythmias and the prevention of sudden cardiac death. *European Heart Journal*.

[13] Chia YMF, Teng THK, Tan ESJ, Tay WT, Richards AM, Chin CWL (2017). Disparity Between Indications for and Utilization of Implantable Cardioverter Defibrillators in Asian Patients With Heart Failure. *Circulation. Cardiovascular Quality and Outcomes*.

[14] Szél T, Koncz I, Antzelevitch C (2013). Cellular mechanisms underlying the effects of milrinone and cilostazol to suppress arrhythmogenesis associated with Brugada syndrome. *Heart Rhythm*.

[15] Shenthar J, Chakali SS, Acharya D, Parvez J, Banavalikar B (2017). Oral quinine sulfate for the treatment of electrical storm and prevention of recurrent shocks in Brugada syndrome after failed cilostazol therapy. *HeartRhythm Case Reports*.

[16] Abud A, Bagattin D, Goyeneche R, Becker C (2006). Failure of cilostazol in the prevention of ventricular fibrillation in a patient with Brugada syndrome. *Journal of Cardiovascular Electrophysiology*.

[17] Tsuchiya T, Ashikaga K, Honda T, Arita M (2002). Prevention of ventricular fibrillation by cilostazol, an oral phosphodiesterase inhibitor, in a patient with Brugada syndrome. *Journal of Cardiovascular Electrophysiology*.

[18] Ağaç MT, Erkan H, Korkmaz L (2014). Conversion of Brugada type I to type III and successful control of recurrent ventricular arrhythmia with cilostazol. *Archives of Cardiovascular Diseases*.

[19] Inoue T, Sohma R, Morooka S (1999). Cilostazol inhibits the expression of activation-dependent membrane surface glycoprotein on the surface of platelets stimulated in vitro. *Thrombosis Research*.

[20] Schrör K (2002). The pharmacology of cilostazol. *Diabetes, Obesity & Metabolism*.

[21] Mallikaarjun S, Forbes WP, Bramer SL (1999). Effect of renal impairment on the pharmacokinetics of cilostazol and its metabolites. *Clinical Pharmacokinetics*.

[22] Bramer SL, Forbes WP (1999). Effect of hepatic impairment on the pharmacokinetics of a single dose of cilostazol. *Clinical Pharmacokinetics*.

[23] Hiatt WR, Money SR, Brass EP (2008). Long-term safety of cilostazol in patients with peripheral artery disease: the CASTLE study (Cilostazol: A Study in Long-term Effects). *Journal of Vascular Surgery*.

[24] Cone J, Wang S, Tandon N, Fong M, Sun B, Sakurai K (1999). Comparison of the effects of cilostazol and milrinone on intracellular cAMP levels and cellular function in platelets and cardiac cells. *Journal of Cardiovascular Pharmacology*.

[25] Liu Y, Fong M, Cone J, Wang S, Yoshitake M, Kambayashi J (2000). Inhibition of adenosine uptake and augmentation of ischemia-induced increase of interstitial adenosine by cilostazol, an agent to treat intermittent claudication. *Journal of Cardiovascular Pharmacology*.

[26] Minami N, Suzuki Y, Yamamoto M, Kihira H, Imai E, Wada H (1997). Inhibition of shear stress-induced platelet aggregation by cilostazol, a specific inhibitor of cGMP-inhibited phosphodiesterase, in vitro and ex vivo. *Life Sciences*.

[27] Elam MB, Heckman J, Crouse JR, Hunninghake DB, Herd JA, Davidson M (1998). Effect of the novel antiplatelet agent cilostazol on plasma lipoproteins in patients with intermittent claudication. *Arteriosclerosis, Thrombosis, and Vascular Biology*.

[28] Rizzo M, Corrado E, Patti AM, Rini GB, Mikhailidis DP (2011). Cilostazol and atherogenic dyslipidemia: a clinically relevant effect?. *Expert Opinion on Pharmacotherapy*.

[29] Kayanoki Y, Che W, Kawata S, Matsuzawa Y, Higashiyama S, Taniguchi N (1997). The effect of cilostazol, a cyclic nucleotide phosphodiesterase III inhibitor, on heparin-binding EGF-like growth factor expression in macrophages and vascular smooth muscle cells. *Biochemical and Biophysical Research Communications*.

[30] Tsuchikane E, Fukuhara A, Kobayashi T, Kirino M, Yamasaki K, Kobayashi T (1999). Impact of cilostazol on restenosis after percutaneous coronary balloon angioplasty. *Circulation*.

[31] Cleanthis M, Bhattacharya V, Smout J, Ashour H, Stansby G (2009). Combined aspirin and cilostazol treatment is associated with reduced platelet aggregation and prevention of exercise-induced platelet activation. *European Journal of Vascular and Endovascular Surgery: the Official Journal of the European Society for Vascular Surgery*.

[32] Hoshino H, Toyoda K, Omae K, Ishida N, Uchiyama S, Kimura K (2021). Dual Antiplatelet Therapy Using Cilostazol With Aspirin or Clopidogrel: Subanalysis of the CSPS.com Trial. *Stroke*.

[33] Kim SM, Jung JM, Kim BJ, Lee JS, Kwon SU (2019). Cilostazol Mono and Combination Treatments in Ischemic Stroke: An Updated Systematic Review and Meta-Analysis. *Stroke*.

[34] Kalantzi K, Tentolouris N, Melidonis AJ, Papadaki S, Peroulis M, Amantos KA (2021). Efficacy and Safety of Adjunctive Cilostazol to Clopidogrel-Treated Diabetic Patients With Symptomatic Lower Extremity Artery Disease in the Prevention of Ischemic Vascular Events. *Journal of the American Heart Association*.

[35] Chen Y, Zhang Y, Tang Y, Huang X, Xie Y (2014). Long-term clinical efficacy and safety of adding cilostazol to dual antiplatelet therapy for patients undergoing PCI: a meta-analysis of randomized trials with adjusted indirect comparisons. *Current Medical Research and Opinion*.

[36] Tang YD, Wang W, Yang M, Zhang K, Chen J, Qiao S (2018). Randomized Comparisons of Double-Dose Clopidogrel or Adjunctive Cilostazol Versus Standard Dual Antiplatelet in Patients With High Posttreatment Platelet Reactivity: Results of the CREATIVE Trial. *Circulation*.

[37] Fujitani K, Kambayashi J, Murata K, Yano Y, Shinozaki K, Yukawa M (1995). Clinical evaluation on combined administration of oral prostacyclin analogue beraprost and phosphodiesterase inhibitor cilostazol. *Pharmacological Research*.

[38] Liang X, Wang Y, Zhao C, Cao Y (2022). Systematic review the efficacy and safety of cilostazol, pentoxifylline, beraprost in the treatment of intermittent claudication: A network meta-analysis. *PloS One*.

[39] Suri A, Bramer SL (1999). Effect of omeprazole on the metabolism of cilostazol. *Clinical Pharmacokinetics*.

[40] Mallikaarjun S, Bramer SL (1999). Effect of cilostazol on the pharmacokinetics and pharmacodynamics of warfarin. *Clinical Pharmacokinetics*.

[41] Suri A, Forbes WP, Bramer SL (1999). Effects of CYP3A inhibition on the metabolism of cilostazol. *Clinical Pharmacokinetics*.

[42] Brugada P, Brugada J (1992). Right bundle branch block, persistent ST segment elevation and sudden cardiac death: a distinct clinical and electrocardiographic syndrome. A multicenter report. *Journal of the American College of Cardiology*.

[43] Wilde AAM, Amin AS (2018). Clinical Spectrum of SCN5A Mutations: Long QT Syndrome, Brugada Syndrome, and Cardiomyopathy. *JACC. Clinical Electrophysiology*.

[44] Hu D, Barajas-Martínez H, Pfeiffer R, Dezi F, Pfeiffer J, Buch T (2014). Mutations in SCN10A are responsible for a large fraction of cases of Brugada syndrome. *Journal of the American College of Cardiology*.

[45] Fukuyama M, Ohno S, Makiyama T, Horie M (2016). Novel SCN10A variants associated with Brugada syndrome. *Europace: European Pacing, Arrhythmias, and Cardiac Electrophysiology: Journal of the Working Groups on Cardiac Pacing, Arrhythmias, and Cardiac Cellular Electrophysiology of the European Society of Cardiology*.

[46] Riuró H, Beltran-Alvarez P, Tarradas A, Selga E, Campuzano O, Vergés M (2013). A missense mutation in the sodium channel β2 subunit reveals SCN2B as a new candidate gene for Brugada syndrome. *Human Mutation*.

[47] Giudicessi JR, Ye D, Tester DJ, Crotti L, Mugione A, Nesterenko VV (2011). Transient outward current (I(to)) gain-of-function mutations in the KCND3-encoded Kv4.3 potassium channel and Brugada syndrome. *Heart Rhythm*.

[48] Burashnikov E, Pfeiffer R, Barajas-Martinez H, Delpón E, Hu D, Desai M (2010). Mutations in the cardiac L-type calcium channel associated with inherited J-wave syndromes and sudden cardiac death. *Heart Rhythm*.

[49] Meregalli PG, Wilde AAM, Tan HL (2005). Pathophysiological mechanisms of Brugada syndrome: depolarization disorder, repolarization disorder, or more?. *Cardiovascular Research*.

[50] Wilde AAM, Postema PG, Di Diego JM, Viskin S, Morita H, Fish JM (2010). The pathophysiological mechanism underlying Brugada syndrome: depolarization versus repolarization. *Journal of Molecular and Cellular Cardiology*.

[51] Alings M, Wilde A (1999). “Brugada” syndrome: clinical data and suggested pathophysiological mechanism. *Circulation*.

[52] Kanlop N, Chattipakorn S, Chattipakorn N (2011). Effects of cilostazol in the heart. *Journal of Cardiovascular Medicine (Hagerstown, Md.)*.

[53] Krittayaphong R, Veerakul G, Nademanee K, Kangkagate C (2003). Heart rate variability in patients with Brugada syndrome in Thailand. *European Heart Journal*.

[54] Wichter T, Matheja P, Eckardt L, Kies P, Schäfers K, Schulze-Bahr E (2002). Cardiac autonomic dysfunction in Brugada syndrome. *Circulation*.

[55] Hernandez OM, Housmans PR, Potter JD (2001). Invited Review: pathophysiology of cardiac muscle contraction and relaxation as a result of alterations in thin filament regulation. *Journal of Applied Physiology (Bethesda, Md.: 1985)*.

[56] Tibbits GF, Hamman BN (1991). Regulation of myocardial contractility. *Medicine and Science in Sports and Exercise*.

[57] Teodorovich N, Kogan Y, Paz O, Swissa M (2016). Vagally mediated ventricular arrhythmia in Brugada syndrome. *HeartRhythm Case Reports*.

[58] Yan GX, Antzelevitch C (1999). Cellular basis for the Brugada syndrome and other mechanisms of arrhythmogenesis associated with ST-segment elevation. *Circulation*.

[59] Shintani S, Watanabe K, Kawamura K, Mori T, Tani T, Toba Y (1985). General pharmacological properties of cilostazol, a new antithrombotic drug. Part II: Effect on the peripheral organs. *Arzneimittel-Forschung*.

[60] Lee KL, Lau CP, Tse HF, Wan SH, Fan K (2000). Prevention of ventricular fibrillation by pacing in a man with Brugada syndrome. *Journal of Cardiovascular Electrophysiology*.

[61] Iqbal M, Aditya Lesmana M, Putra ICS, Karwiky G, Achmad C, Goenawan H (2024). Implications of Associated Atrial Fibrillation in Brugada Syndrome for Sudden Cardiac Death - A Case Series Analysis. *The American Journal of Case Reports*.

[62] Letsas KP, Sideris A, Efremidis M, Pappas LK, Gavrielatos G, Filippatos GS (2007). Prevalence of paroxysmal atrial fibrillation in Brugada syndrome: a case series and a review of the literature. *Journal of Cardiovascular Medicine (Hagerstown, Md.)*.

[63] Chen LY, Benditt DG, Alonso A (2014). Atrial fibrillation and its association with sudden cardiac death. *Circulation Journal: Official Journal of the Japanese Circulation Society*.

[64] Chugh SS, Reinier K, Teodorescu C, Evanado A, Kehr E, Al Samara M (2008). Epidemiology of sudden cardiac death: clinical and research implications. *Progress in Cardiovascular Diseases*.

[65] Micaglio E, Monasky MM, Ciconte G, Vicedomini G, Conti M, Mecarocci V (2019). Novel SCN5A Frameshift Mutation in Brugada Syndrome Associated With Complex Arrhythmic Phenotype. *Frontiers in Genetics*.

[66] Aoki J, Iguchi Y, Urabe T, Yamagami H, Todo K, Fujimoto S (2020). Cilostazol uncovers covert atrial fibrillation in non-cardioembolic stroke. *Journal of the Neurological Sciences*.

[67] Priori SG, Napolitano C, Gasparini M, Pappone C, Della Bella P, Brignole M (2000). Clinical and genetic heterogeneity of right bundle branch block and ST-segment elevation syndrome: A prospective evaluation of 52 families. *Circulation*.

[68] Chen Q, Kirsch GE, Zhang D, Brugada R, Brugada J, Brugada P (1998). Genetic basis and molecular mechanism for idiopathic ventricular fibrillation. *Nature*.

[69] Patruno N, Pontillo D, Achilli A, Ruggeri G, Critelli G (2003). Electrocardiographic pattern of Brugada syndrome disclosed by a febrile illness: clinical and therapeutic implications. *Europace: European Pacing, Arrhythmias, and Cardiac Electrophysiology: Journal of the Working Groups on Cardiac Pacing, Arrhythmias, and Cardiac Cellular Electrophysiology of the European Society of Cardiology*.

[70] Mok NS, Priori SG, Napolitano C, Chan NY, Chahine M, Baroudi G (2003). A newly characterized SCN5A mutation underlying Brugada syndrome unmasked by hyperthermia. *Journal of Cardiovascular Electrophysiology*.

